# Genome-Wide Identification of the *Alba* Gene Family in Plants and Stress-Responsive Expression of the Rice *Alba* Genes

**DOI:** 10.3390/genes9040183

**Published:** 2018-03-28

**Authors:** Jitendra Kumar Verma, Vijay Wardhan, Deepali Singh, Subhra Chakraborty, Niranjan Chakraborty

**Affiliations:** 1 National Institute of Plant Genome Research, Jawaharlal Nehru University Campus, Aruna Asaf Ali Marg, New Delhi 110067, India; jitendra20020@gmail.com (J.K.V.); v_wardhan@yahoo.co.in (V.W.); subhrac@hotmail.com (S.C.); 2 School of Biotechnology, Gautam Buddha University, Greater NOIDA, Gautam Budh Nagar, Uttar Pradesh 201308, India; deepali@gbu.ac.in

**Keywords:** Alba domain, architectural proteins, evolutionary relevant, phylogenetic relationship, regulatory elements, subcellular localization, 3D structure

## Abstract

Architectural proteins play key roles in genome construction and regulate the expression of many genes, albeit the modulation of genome plasticity by these proteins is largely unknown. A critical screening of the architectural proteins in five crop species, viz., *Oryza sativa*, *Zea mays*, *Sorghum bicolor*, *Cicer arietinum*, and *Vitis vinifera*, and in the model plant *Arabidopsis thaliana* along with evolutionary relevant species such as *Chlamydomonas reinhardtii*, *Physcomitrella patens*, and *Amborella trichopoda*, revealed 9, 20, 10, 7, 7, 6, 1, 4, and 4 *Alba* (acetylation lowers binding affinity) genes, respectively. A phylogenetic analysis of the genes and of their counterparts in other plant species indicated evolutionary conservation and diversification. In each group, the structural components of the genes and motifs showed significant conservation. The chromosomal location of the *Alba* genes of rice (*OsAlba*), showed an unequal distribution on 8 of its 12 chromosomes. The expression profiles of the *OsAlba* genes indicated a distinct tissue-specific expression in the seedling, vegetative, and reproductive stages. The quantitative real-time PCR (qRT-PCR) analysis of the *OsAlba* genes confirmed their stress-inducible expression under multivariate environmental conditions and phytohormone treatments. The evaluation of the regulatory elements in 68 *Alba* genes from the 9 species studied led to the identification of conserved motifs and overlapping microRNA (miRNA) target sites, suggesting the conservation of their function in related proteins and a divergence in their biological roles across species. The 3D structure and the prediction of putative ligands and their binding sites for OsAlba proteins offered a key insight into the structure–function relationship. These results provide a comprehensive overview of the subtle genetic diversification of the *OsAlba* genes, which will help in elucidating their functional role in plants.

## 1. Introduction

Plants encounter multiple abiotic and biotic stresses in a complex environment, which hamper their growth and development [1]. The stress perception and transduction of signals activate self-defense mechanisms in plants for acclimatization and survival by alterations in protein balance. The regulation of gene expression differs widely between prokaryotes and eukaryotes. The evolutionarily conserved DNA-binding proteins, particularly histones in the chromatin, act as an on-off switch that turns genes on or off [2,3]. The DNA-binding Alba (acetylation lowers binding affinity) superfamily of proteins has received global attention immediately after the Alba proteins were identified from archaea as sequence-independent DNA-binding proteins [4,5,6]. The members of this superfamily are diversely found in archaea and eukaryotes as small, basic, dimeric nucleic acid-binding proteins [7,8,9,10,11]. Although Alba proteins behave as histone-like DNA-binding components, RNA-binding properties of Alba have also been reported [4,6,12,13]. The proteins of the Alba family regulate gene expression through acetylation–deacetylation and have essentially an Alba domain. Recognized as chromosomal proteins, these are speculated to help in maintaining chromatin architecture and transcriptional repression. Nevertheless, the functional diversity among Alba proteins encompasses transcriptional and translational regulation, genome packaging and organization, development and differentiation, and RNA metabolism [2,8,11,14]. The biological roles of Alba proteins are poorly understood because of their multiple paralogs, posttranslational modifications, differential binding affinity, and functional crosstalk. 

The structure and function of a protein are strongly related to the degree to which an amino acid site is free to vary, and such function can be predicted by identifying the target sites for positive selection in the course of evolution. The eukaryotic Alba homologs are allocated in two families including the Rpp20/Pop7 subunit of RNaseP/MRP orthologues in humans, *Caenorhabditis elegans*, yeast, RNA-binding proteins in protozoans, rice, and *Arabidopsis*, while the second family comprises the Rpp25 subunit of RNaseP, ciliate Mdp2, and related proteins in protozoans and fruit fly [4]. Among the four Alba proteins identified in *Plasmodium falciparum* [14,15], the restriction of PfAlba5 and PfAlba6 to parasitic protozoa indicates their lineage-specific evolution [16]. In *Trypanosoma brucei*, four Alba family proteins (TbAlba1, TbAlba2, TbAlba3, and TbAlba4) have been recognized [17,18,19]. Most Alba proteins present a generic single-domain architecture (826 sequences), while the rest shows multidomain architectures (18 sequences) [20]. Other domains, which are present along with the Alba-domain, include the CLIP1 zinc finger domain in the nematode *Pristionchus*, the subunit p22 domain of dynactin (Dynactin p22), the FAD-binding domain, the F-box domain in the fungus *Taphrina*, the NT5C in the oomycote *Pythium*, the ATP synthase subunit H domain in the protozoan parasite *Thileria*, and the sugar transporter domain in the Florida lancelet [21]. Moreover, a C-terminal RGG box has been found in Alba3 and Alba4 in *T. brucei* [17], in an Alba-domain protein in *Leishmania infantum* [22], in Alba1 in *Toxoplasma gondii* [23], and in Alba1 and Alba2 in *P. falciparum* [14] and *Plasmodium berghei* [24]. The RGG box has been implicated in DNA damage signalling, regulation of apoptosis, transcription, pre-mRNA splicing, and translation [25]. Interestingly, plant-specific Alba proteins demonstrate utmost diversity in their domain architecture. These proteins harbour various other domains, viz., proteasome regulatory subunit, xylanase inhibitor N-terminal (Taxi_N), thioredoxin-4 (Trx-4), and DEAD/DEAH box helicase domains in combination with an Alba domain [21]. The speciation and functional divergence in Alba proteins might be attributed to the emergence of novel domain combinations with the Alba domains. The evolution of multidomain combinations in Alba proteins might be a case of domain recombination, duplication, and divergence, which influence their DNA- and RNA-binding properties as well as other cellular processes.

We investigated the contribution of Alba proteins harbouring an Alba domain (Pfam PF01918) and having nucleic acid–binding activity. Further, the rice *Alba* genes, *OsAlba*, were identified and characterized by chromosome location, integration of sequence features, evolutionary origin, and phylogenetic relationship, besides their stress-responsive expression. These results may provide new insights into the evolutionary relationship of plant-specific *Alba* genes and their biological relevance. 

## 2. Materials and Methods

### 2.1. Identification and Sequence Retrieval of *Alba* Genes

The whole genome sequence of plants, including *Chlamydomonas* (a green algae), *Physcomitrella* (a bryophyte), and *Amborella* (basal angiosperm), along with monocot (rice, maize. and sorghum) and dicot (grape, chickpea, and *Arabidopsis*) representatives were retrieved from the annotation databases (DB) Phytozome v12.0 [26] and NCBI. The Pfam database [27] was used to retrieve the Hidden Markov Model (HMM) profile of the Alba domain (Pfam PF01918), which was further submitted to BLASTP (*p* = 0.001) search. The amino acid sequences were examined for the presence of the Alba domain using the NCBI Conserved Domain Database (CDD) [28]. The Alba protein sequences of rice and *Arabidopsis* were independently aligned with those of chickpea, maize, and sorghum using BLASTP (*e*-value cutoff: 1 × 10^−5^). The HMM profiles obtained from Pfam database for the Alba domain and the putative *Alba* genes identified from other species were merged to develop a non-redundant list for each species and were examined for the presence of a conserved Alba domain.

### 2.2. Sequence Analysis and Structural Characterization

The information regarding protein sequences, genomic sequences, coding DNA sequences (CDS), and upstream 1500-bp nucleotide stretches from the translation initiation codon along with their locations on the chromosomes were downloaded (www.phytozome.net). To analyze the structure and diversity of the *Alba* genes and the exon–intron positions, their sequences were surveyed using the online GSDS2.0 program [29]. The MEME online tools [30] were used to identify the motifs of Alba proteins. The parameters chosen were: maximum length of the conserved motif, 50; minimum length, 6; largest number, 15.

### 2.3. Phylogenetic Analysis

The amino acid sequences of Alba proteins from *Chlamydomonas* (*CreAlba*), *Physcomitrella* (*PpAlba*), *Amborella* (*AmtrAlba*), grape (*VvAlba*), chickpea (*CaAlba*), *Arabidopsis* (*AtAlba*), rice (*OsAlba*), maize (*ZmAlba*), and sorghum (*SbAlba*) were used to construct the cladogram. The SsoAlba1 of *Sulfolobus solfataricus* was used as an outgroup. The multiple sequence alignment (MSA) of the Alba proteins was conducted using Clustal omega. The MEGA 7.0 software was used with default settings to construct a neighbor-joining phylogenetic tree [31]. The statistical significance for each tree node was determined by bootstrap analysis using 100 replicates [32].

### 2.4. Subcellular Localization and Analysis of Cis-Acting Regulatory Elements in *OsAlba*

The subcellular localization was analyzed in silico using BaCelLo [33], ESLPred [34], YLoc [35], PSORT II [36], LocTree3 [37], WoLF PSORT [38], Plant-mPLoc [39], and CELLO [40]. The NCBI database was used to retrieve a 1500-bp DNA sequence from the 5′-upstream region of each *Alba* gene, and the corresponding *cis*-acting regulatory elements were investigated with the plantCARE database [41]. 

### 2.5. Prediction of miRNA Targets

The putative miRNA targets were identified with the psRNATarget program [42] using default parameters. Recently identified miRNA and considerably different known miRNA sequences were used as custom sequences. The redundant sequences were removed after identifying potential target mRNA sequences for further analysis.

### 2.6. Molecular Modeling of OsAlba Proteins

The 3D structure of the rice Alba proteins was generated by I-TASSER server [43]. The sequences of OsAlba1–9 were used as input. The 3D models were developed from multiple threading alignments and iterative structural assembly simulations. We chose the best model with the highest scores, retrieved the template analogs, predicted the ligand-binding sites, and refined the model with PyMOL software v1.3. The secondary and tertiary structures were further analyzed for predicting the ligand-binding sites and GO terms. 

### 2.7. Plant Materials, Growth Conditions, and Stress Treatment

The seedlings of rice (*Oryza sativa* L. ssp. *indica*) were grown in pots containing a combination of soilrite and soil (1:2, *w/w*; 10–12 seedlings/pot) in a program-regulated growth chamber. The seedlings were maintained at 70 ± 5% relative humidity under a 16 h photoperiod (300 µmol∙m^−2^·s^−1^ light intensity) at 28 ± 2 °C. Two-week-old seedlings were exposed to multivariate stresses (dehydration, hypersalinity, high temperature, and cold) as described earlier [44]. In a separate set, the seedlings were supplied with 200 mM NaCl solution, and the samples were collected at 0, 6, 12, and 24 h. The hormonal treatments were carried out by spraying the seedlings with abscisic acid (ABA, 200 µM), jasmonic acid (JA, 200 µM), and salicylic acid (SA, 200 µM), and the tissues were harvested at 0, 0.5, 1, 3, 6, and 12 h intervals [44]. Independently, the seedlings were kept at 4 °C for cold stress and sampled at 0, 1, 3, 6, and 12 h post-treatment. Dehydration was imposed by withholding water supply, and the tissues were collected at 0-, 2-, 4-, and 5-day intervals. High temperature treatment was imposed by transferring the potted seedlings to 42 °C chamber, harvesting at 0, 6, 12, and 24 h post-treatment. For tissue-specific expression analysis, the tissues were collected from six major organs, viz., roots, stems, leaves, flag leaves, leaf sheath, and panicles of 120-day-old plants. A total of three replicates were chosen for each experiment (at an average of three plants per replica). The tissues sampled for each treatment were frozen in liquid nitrogen and stored at −80 °C, unless otherwise mentioned. The tissues from the unstressed seedlings were harvested for each time point for various stress treatments and finally pooled to normalize any possible effect of growth and development.

### 2.8. RNA Isolation and Quantitative Real-Time PCR Analysis

Total RNA was isolated using TRI reagent (Sigma Life Science, St. Louis, MO, USA) as per the manufacturer’s instructions. The quantity and quality of the extracted RNA was estimated using Nanodrop1000 (Thermo Fisher Scientific, Wilmington, DE, USA). The complementary DNA (cDNA) was synthesized using RNA samples with a value of 260/280 ratio between 1.8 and 2.1, and 260/230 ratio between 2.0 and 2.5. The gene-specific primers were designed using the Primer Express software v3.0 (Applied Biosystems, Foster City, CA, USA) and are listed in Table S1. Two biological replicates from each treatment, comprised of at least three technical replicates, were analyzed. The transcript analysis was conducted by quantitative real-time PCR (qRT-PCR) using an ABI Prism 7500 Detection System (Applied Biosystems, Foster City, CA, USA). The reactions were carried out in a 10 μL volume containing 200 nM primers, 5 μL SYBR Premix Ex Taq II (Takara, Beijing, China), and 1 μL diluted cDNA in the following conditions: 10 min at 95 °C, 40 cycles of 15 s at 95 °C, and 30 s at 60 °C. The melting curve analysis was carried out to verify the specificity of the amplicon for each primer pair. The expression of tubulin was used as an internal control for normalization.

## 3. Results and Discussion

### 3.1. Identification of Plant-Specific Alba Proteins

The identification and in silico analysis of the Alba superfamily was carried out with the available genome sequences. The gene family was investigated following two strategies, i.e., HMM profile search and BLASTP. A non-redundant list was obtained for rice and maize by combining the identified *Alba* genes. To further confirm the conserved Alba domain, Pfam and SMART databases were used for the candidate proteins. The analysis revealed 1, 4, 4, 7, 7, 6, 9, 20. and 10 *Alba* genes in *Chlamydomonas*, *Physcomitrella*, *Amborella*, grape, chickpea, *Arabidopsis*, rice, maize, and sorghum, respectively. The presence of *Alba* genes as a multigene family in higher eukaryotes suggests their biological significance. The characteristic signatures of *CreAlba*, *PpAlba*, *AmtrAlba*, *VvAlba*, *CaAlba*, *AtAlba*, *OsAlba*, *ZmAlba*, and *SbAlba* representing gene names, identifier, chromosome location, mRNA length, CDS, and protein sequence along with their physical and chemical properties showed a significant conservation as well as variations (Table 1).

### 3.2. Genomic Organization of *Alba* Genes and Their Chromosomal Distribution

**Figure 1:**
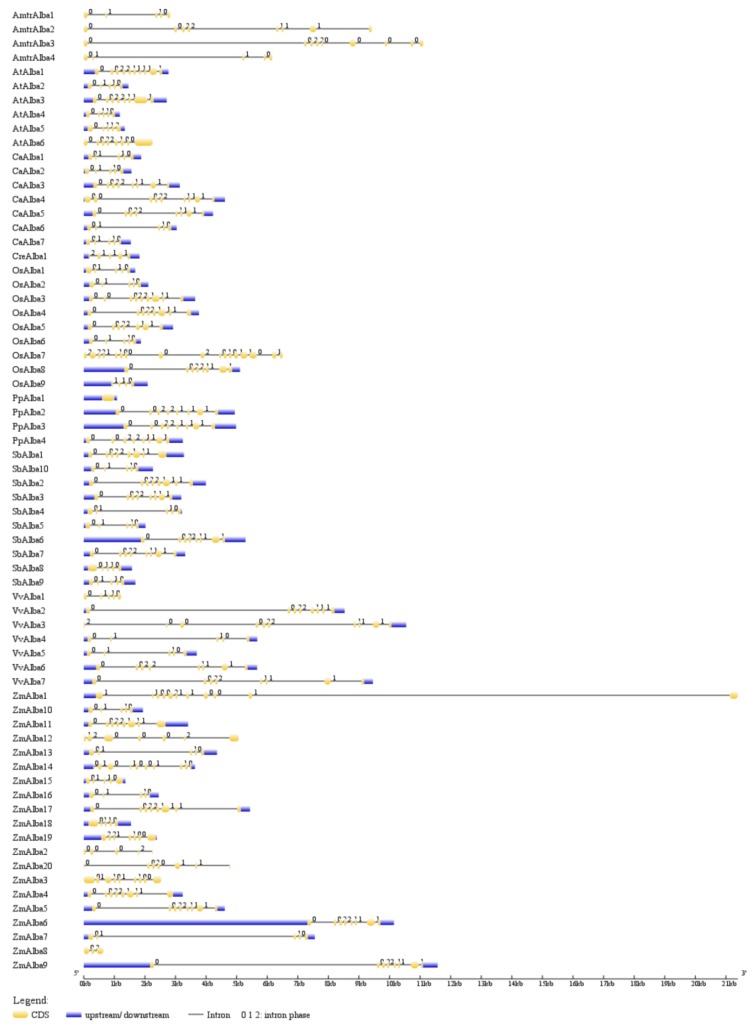
Structural organization of plant-specific *Alba* (acetylation lowers binding affinity) genes. Exons and introns in the genes are represented by boxes and lines, respectively. Intron phase 0, phase 1, and phase 2 are indicated.

The Alba proteins identified in monocots and dicots showed the characteristic Alba domain with sequence conservation in the core region. The exon–intron organization of the plant-specific *Alba* genes revealed variation in the intron number ranging 0–17. Most of the *Alba* genes had a similar intron-phasing distribution (Figure 1). 

Next, we analyzed the chromosomal distribution of the *Alba* genes. In rice, the *Alba* genes were found to be distributed unevenly throughout the chromosomes. All nine *OsAlba* genes studied were found to be distributed on the 12 chromosomes, except for chromosomes 5, 7, 8, and 10. However, the number of *Alba* genes varied widely on each chromosome. Most of the *Alba* genes were found to be localized on the distal ends of the chromosomes. Two genes were found to be located on chromosome 3, whereas one each were identified on chromosomes 1, 2, 4, 6, 9, 11, and 12. In maize, all 20 *ZmAlba* genes were found to be distributed on chromosomes 1–10: 4 located on chromosome 1, 3 each on chromosomes 8 and 10, 2 each on chromosomes 2, 3, 4, and 9, while 1 each on chromosomes 5 and 7 (Table 1). In sorghum, *SbAlbas* genes were found to be distributed on all the chromosomes: two on chromosome 1, while one each on chromosomes 2, 3, 4, 5, 6, 8, 9, and 10. We observed seven *Alba* genes in chickpea with three genes on chromosome 5 and one each located on chromosomes 1, 4, 6, and 8. In grape, there were seven genes distributed on chromosomes 5, 7, 9, 11, 18. In *Arabidopsis*, all the six *AtAlba* genes were found to be distributed on three of five chromosomes: three were present on chromosome 1, two on chromosome 3, and one on chromosome 2. In *Chlamydomonas*, there was only one *Alba* gene located on chromosome 9. In *Physcomitrella*, all four *Alba* genes were distributed on chromosomes 12, 20, 23, 24. In *Amborella*, all the *Alba* genes were found to be present on the scaffold00002, scaffold00017, scaffold00067, and scaffold00104. The major forces during the course of genome evolution in plants have been the duplication of individual genes, of chromosomal segments, or of the entire genome itself [45]. We analyzed the possibility of gene duplication in the *Alba* gene family using the Plant Genome Duplication database [46]. The distribution of *OsAlba1* and *OsAlba6* on chromosomes 1 and 6, respectively, suggests a segmental duplication. Similarly, *ZmAlba7* and *ZmAlba13* on chromosomes 1 and 6 and *ZmAlba4* and *ZmAlba11* on chromosomes 1 and 5 indicate gene duplication. The presence of more than one member of a gene family on the same chromosome is suggestive of tandem duplication, while segmental duplication is defined as the event of gene duplication on different chromosomes [47]. In sorghum, tandem duplication was observed in *SbAlba1* and *SbAlba2* on chromosome 1, while segmental duplication was observed in *SbAlba4*, *SbAlba9*, and *SbAlba10* on chromosomes 3, 9, and 10, respectively. Interestingly, the presence of *SbAlba4*, *SbAlba5*, and *SbAlba10* on chromosomes 3, 4, and 10, respectively, was also predicted to be an event of segmental duplication. *Arabidopsis* showed tandem duplication for *AtAlba1* and *AtAlba2* on chromosome 1. These results suggest a tandem as well as a segmental duplication across the *Alba* gene family in plants. The variation in gene sequences during duplication indicated the neofunctionalisation of the paralogs [48]. It is suggested that two genes with identical functions can stably be maintained in the genome only when an extra amount of a gene product becomes advantageous for an organism [49]. To investigate the positive selection among OsAlba proteins, we analyzed the value of ω (dN/dS) through the codeml programme of PAML software by the maximum-likelihood method [50]. While OsAlba2 and OsAlba3 indicated a high non-synonymous substitution rate, OsAlba2 and OsAlba4 along with OsAlba4 and OsAlba6 suggested substitutions in an adaptive manner. The expansion of the *Alba* gene family in plants through evolution provides new insights into their diverse biological roles.

### 3.3. Phylogenetic Analysis of *Alba* Gene Families

**Figure 2:**
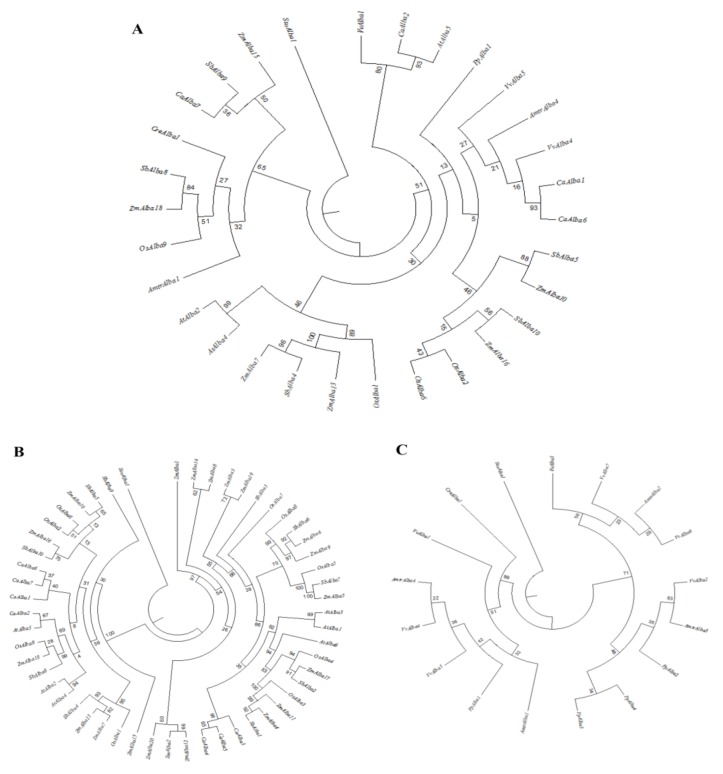
Phylogenetic relationships of plant-specific Alba proteins. (**A**) Phylogenetic tree constructed using the most conserved region of the Alba domain from the closest Alba homologs across the species studied; (**B**) phylogenetic relationships of all Alba homologs from rice, maize, sorghum, chickpea, and *Arabidopsis*; (**C**) phylogenetic relationships of all Alba homologs from *Chlamydomonas*, *Physcomitrella*, *Amborella*, and grape. The analysis was carried out with the maximum-likelihood method with the bootstrap values from 100 replicates, as indicated by the numerical values on the nodes.

To explore the evolutionary relationships among various Alba family members, full-length amino acid sequences were analyzed. The MSA of sequences was performed, and, sequentially, the phylograms were generated (Figure 2).

**Figure 3:**
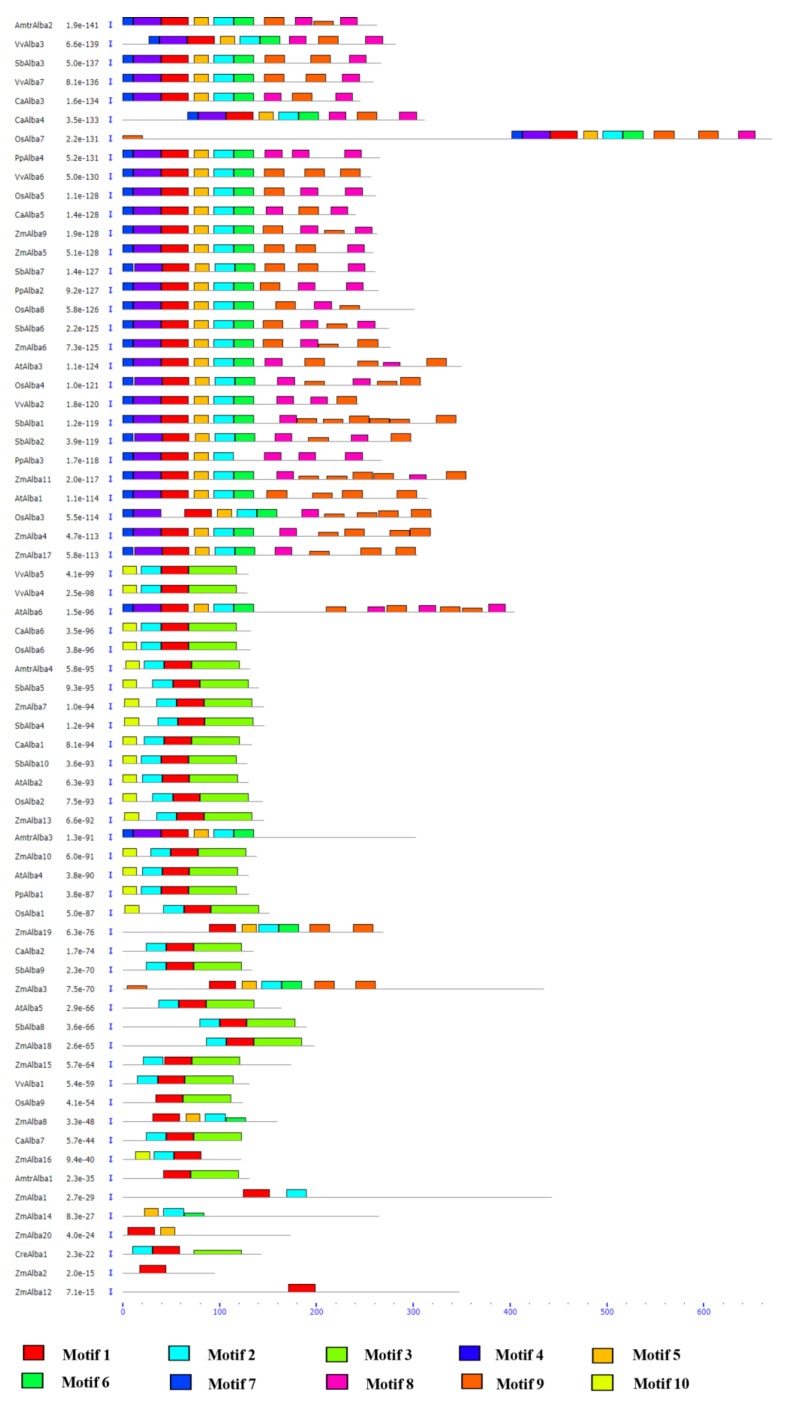
Schematic representation of the conserved motifs in plant-specific Alba proteins. The motifs in the proteins are represented by colored boxes, with the motif name indicated on the right. The length of the boxes represents the estimated motif length.

Three different phylogenetic trees were constructed using the Alba proteins from the crop families and Alba proteins from other species to better understand the phylogenetic relationships. The phylogram constructed with the most conserved region of the Alba domain of the most similar Alba homologs across species indicated a major grouping between dicots and monocots along with lower plants (Figure 2A). The analysis with all the Alba homologs from the crop species showed two major clades from monocots and dicots. Proteins from rice, maize, and sorghum clustered together, while proteins from chickpea and *Arabidopsis* formed separate groups (Figure 2B). The Alba proteins from *Chlamydomonas*, *Amborella*, *Physcomitrella*, and grape, showed diversity in sequences as compared to most of their homologs in the crop species (Figure 2C). Interestingly, the Alba proteins of grape and *Amborella* were found to be clustered together, while their counterparts from lower plants grouped separately. Altogether, these results suggested diversity in Alba proteins among the different strata of species along with sequence conservation within similar groups. The rice Alba family of proteins revealed the existence of two distinct groups. The first group was found to contain the typical N-terminal Alba domain, while the second group possessed an Alba domain in the middle. However, OsAlba7 showed a C-terminal Alba domain with a different sequence composition compared to other members. Interestingly, the Alba proteins with a similar domain composition were clustered in the same clade. A diverse domain architecture in plant-specific Alba sequences has previously been reported [51]. Proteasome regulatory subunit Rpn3_C, Trx-4, DEAD/DEAH box helicase, Taxi_N, and Taxi_C domains were found in combination with the Alba domain across the species [21]. The phylogenetic analysis grouped *L. infantum* Alba proteins in Rpp25/Mdp2 (*LiAlba20* or *LiAlba3*) and Rpp20/Pop7 (*LiAlba13* or *LiAlba1*) groups [22,52]. In *Trypanosoma*, *TbAlba1* and *TbAlba2* were classified in the Rpp20/Pop7 subunit-containing Alba-domain family, whereas *TbAlba3* and *TbAlba4* were grouped together with the Rpp25/Mdp2 subfamily [16]. Furthermore, the motif analysis led to the identification of 15 different conserved motifs, with a sharing of conserved motifs among related proteins (Figure 3). 

The order, number, and type of motifs were found to be similar in proteins within the same subfamily, but differed across the subfamilies. Motif 1, 2, and 3 were found to be conserved in most Alba proteins analyzed, and motif 4 and 5 were the most conserved among plant-specific Alba proteins (Figure S1). The combination of different domains and motifs along with the Alba domains indicates the possible functional diversities of these proteins across species, as suggested by earlier reports [11,21]. 

### 3.4. Subcellular Localization of the Alba Family Proteins

To analyze the subcellular location, 68 plant-specific Alba proteins were examined using various localization-prediction tools. While the Alba proteins were primarily predicted to be translocated to the nucleus, cytoplasm, or chloroplast, the majority of them indicated their localization in the nucleus (Table S2). The in-silico prediction analysis in this study and our previous observation of OsAlba1 being translocated to the nucleus [53], confirmed the nuclear localization of the rice Alba proteins. A comparison of data from the species studied further validated the nuclear localization of Alba proteins. The nuclear localization of Alba proteins is suggestive of their putative role in gene expression, particularly in stress responses [53]. In archaea, the Alba proteins were previously shown to be involved in histone modifications, besides the regulation of gene expression [54]. 

### 3.5. Analysis of Upstream Regulatory Elements in *Alba* Genes

To evaluate the regulation of plant-specific *Alba* genes under stress conditions, we examined 1500-bp sequences upstream of the transcriptional start site. The identified putative *cis*-acting regulatory elements (CAREs) were classified into seven groups: enhancer, essential element, hormone-responsive, stress-responsive, and other elements (Table S3). The CAREs associated with environmental stress and hormone response included ABRE, CE1, and CE3 involved in ABA response; MYB-binding sites (MBS) involved in water-deficit; low temperature responsive elements (LTRs) involved in the cold and hypersalinity response; TCA-element involved in SA response; and TGACG-motif and CGTCA-motif involved in JA response. These results indicate the stress-responsive role of *Alba* genes across plant species. 

We observed the presence of more than one stress- and hormone-responsive elements in the proximal promoter region of the *Alba* genes. The rice *Alba* genes were found to contain multiple stress-responsive elements (MBS, HSE, LTR, and TC-rich repeats) along with hormone-responsive elements (CGTCA-motif and TGACG-motif). An ABA-responsive motif (ABRE) was detected in all *OsAlba* genes except for *OsAlba7*, suggesting their ABA-mediated regulation. The maize *Alba* genes also showed an ABRE motif as well as other stress- and hormone-responsive elements in the promoter region. Most *Alba* genes in sorghum harbored dehydration- and hormone-responsive motifs, including ABRE elements in *SbAlba1*, *SbAlba2*, *SbAlba4*, *SbAlba5*, and *SbAlba8*. In chickpea, stress- and hormone-responsive motifs were prevalent in *CaAlba3* and *CaAlba4*, while *VvAlba3*, *VvAlba4*, *VvAlba5*, and *VvAlba6* in grape showed the presence of an ABRE motif along with other stress-responsive elements. *Arabidopsis Alba* genes, *AtAlba1*, *AtAlba2*, and *AtAlba3* also showed such elements, besides ABRE and MBS motifs in *AtAlba1-6*. Interestingly, an ABRE motif was found in *Chlamydomonas*, indicating its origin in primitive plants, which might have become functional in higher plants. Along with other stress- and hormone-responsive motifs, an ABRE motif was also found in *PpAlba1* and *PpAlba2* of *Physcomitrella* and in all four *Alba* genes of *Amborella* (Table S3). Altogether, these results suggest that the stress-responsive motif along with ABRE in *Alba* genes might have a key role in stress tolerance.

### 3.6. Prediction of miRNA Targets in Plant-Specific Alba Proteins

Under stress conditions, many genes have been reported to be regulated post-transcriptionally through several miRNA families [55]. We identified miRNA target sequences for miR2673 and few other miRNAs in *Alba* genes (Table S4) using the miRNA prediction tool (http://plantgrn.noble.org/psRNATarget/). More than one target sites for miR2673 was found in *OsAlba5*, *ZmAlba5*, *SbAlba7,* and *AtAlba1*. miR2673 was found to regulate genes responsible for auxin- and ethylene-mediated signal transduction [56], plant defense response, and cellular signaling [57]. This miRNA was also found to be upregulated during the late induction of fruit abscission, senescence, and proline accumulation [58]. A target site for miR5208, which was previously reported to be involved in disease responses, was found in *OsAlba7*, *ZmAlba3*, *ZmAlba14*, *ZmAlba8*, and *SbAlba3* (Table S4) [59]. *ZmAlba1* exhibited a target site for miR444, which was earlier shown to be associated with the cold response [60]. Further, a target site for miR394, which was previously documented in hypersalinity and phytohormone response, was found in *ZmAlba19* [61,62,63].

### 3.7. Tissue-Specific Expression Profiles of *OsAlba* Genes

**Figure 4:**
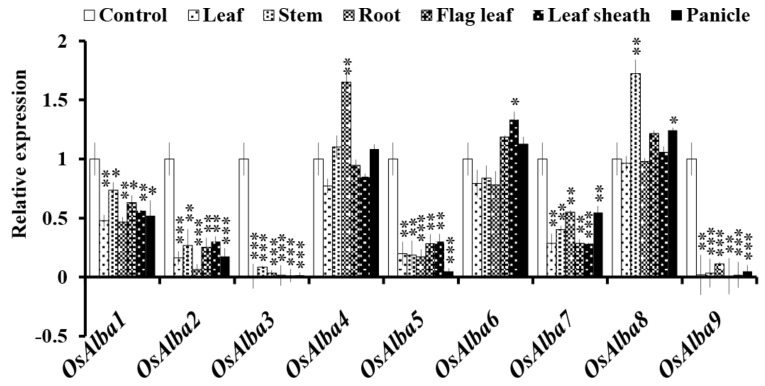
Tissue-specific expression of the *OsAlba* genes. The transcript profile of the *Alba* genes was determined in six major organs, viz., roots, stems, leaves, flag leaves, leaf sheath, and panicle. Germinating seedlings (control) were used as a reference to quantitate the relative mRNA levels in different tissues. The error bars indicate SE (standard error). The asterisk marks indicate a statistically significant difference between the control and other tissues (* *p* ≤ 0.05, ** *p* ≤ 0.01 and *** *p* ≤ 0.001 and **** *p* ≤ 0.0001).

To determine the biological roles of *OsAlba* genes, their transcript abundance was evaluated in six major organs, viz., roots, stems, leaves, flag leaf, leaf sheath, and panicle. While *OsAlba1* and *OsAlba7* showed a moderate expression in all tissues analyzed, *OsAlba2* and *OsAlba5* showed a minimum expression. *OsAlba3* and *OsAlba9* showed a minimum expression or no expression. However, *OsAlba4*, *OsAlba6*, and *OsAlba8* were found to be highly expressed in all tissue types (Figure 4). 

While *OsAlba4* was found to be root-specific, *OsAlba8* was mostly expressed in the stem. Furthermore, *OsAlba4*, *OsAlba6*, and *OsAlba8* showed comparatively higher expression in the flag leaf and panicles. The flag leaf and panicles in rice play an important role in providing photosynthates [64] and help in grain filling during seed development [65]. Additionally, the panicles support the survival of seeds during dehydration and heat stress [66], indicating a vital role of *OsAlba4*, *OsAlba6*, and *OsAlba8* in rice. The differential expression pattern of these genes indicates that they might have a role in the coordination of various physiological pathways.

### 3.8. Stress-Induced Expression of *OsAlba* Genes 

**Figure 5:**
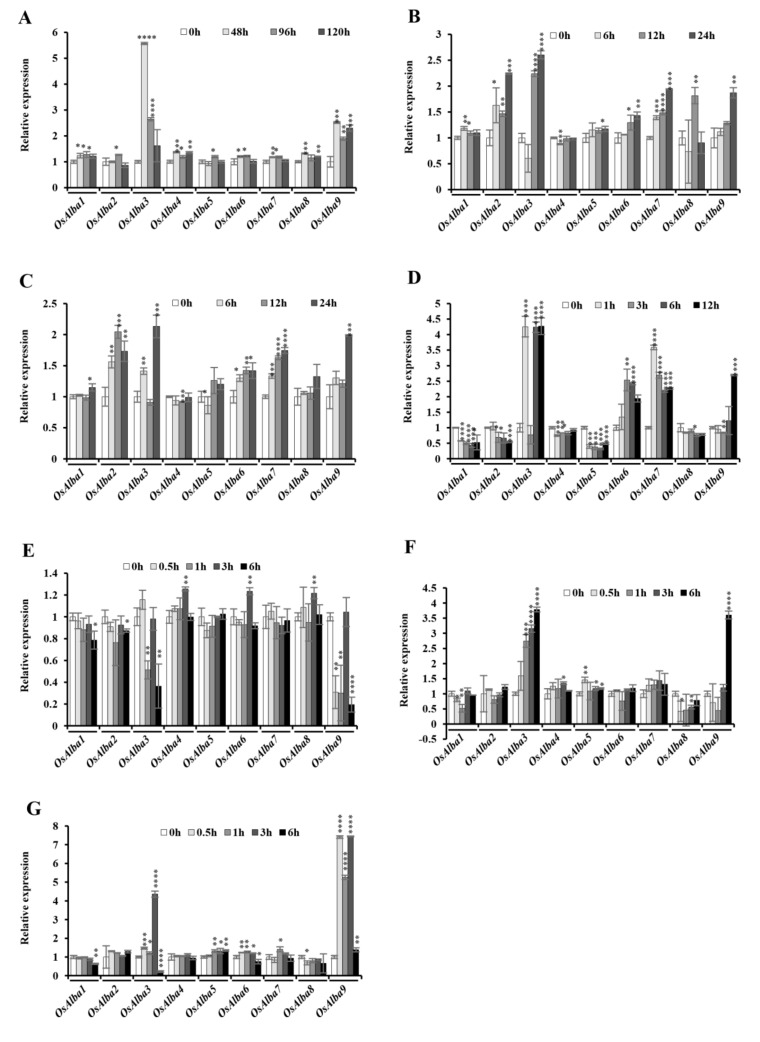
Relative expression of *OsAlba* genes under multivariate stresses. The transcript abundance in seedlings exposed to (**A**) dehydration; (**B**) hypersalinity (200 mM NaCl); (**C**) high temperature (42 °C); (**D**) low temperature (4 °C); treatment with phytohormones (**E**) abscisic acid (ABA, 200 µM/L); (**F**) jasmonic acid (JA, 200 µM/L); and (**G**) salicylic acid (SA, 200 µM/L). The samples were collected at different time points of stress imposition and phytohormone treatments. The asterisk marks denote statistically a significant difference between the control and other tissues (* *p* ≤ 0.05, ** *p* ≤ 0.01, *** *p* ≤ 0.001 and **** *p* ≤ 0.0001).

To gain a deeper insight into the role of *OsAlba* genes in stress tolerance, we investigated their transcript profiles under dehydration, hypersalinity, heat, and cold. The *Alba* genes showed a diverse expression pattern suggesting their stress-induced differential responses. The transcripts of *OsAlba1* were induced significantly under dehydration, heat, and hypersalinity, but showed reduced expression under cold (Figure 5). 

Under dehydration, *OsAlba3*, *OsAlba4*, *OsAlba6*, *OsAlba7*, *OsAlba8*, and *OsAlba9* showed upregulation, *OsAlba4*, *OsAlba7* and *OsAlba9* showed a steady-state level, and *OsAlba5* showed downregulated expression. Under heat and hypersalinity, *OsAlba1*, *OsAlba2*, *OsAlba6*, and *OsAlba7* were upregulated, while *OsAlba4* showed downregulated expression. *OsAlba8* showed induced expression under heat and lower expression under hypersalinity. Under cold stress, *OsAlba3*, *OsAlba6*, and *OsAlba7* were upregulated, but *OsAlba2*, *OsAlba4*, *OsAlba5*, and *OsAlba8* were downregulated. *OsAlba9* showed reduced expression up to 3 h, but its expression was induced subsequently at 6 and 9 h of exposure. The transcript abundance of *OsAlba7* was markedly induced under hypersalinity, cold, heat, and dehydration indicating its role in multivariate stress responses. Interestingly, *OsAlba3* and *OsAlba9* exhibited upregulated expression under dehydration and hypersalinity, suggesting their stress-responsive function. *OsAlba6* and *OsAlba8* showed tissue-specific expression in the flag leaf and panicles (Figure 4) and induced expression during heat stress, indicating their possible role in seed maintenance during stress conditions. Several flag leaf- and panicle-specific genes are known to be induced under dehydration, hypersalinity, and heat stress [66,67], and the overlap of stress-responsive gene expression in different organs is no exception in rice [68]. These results demonstrated that most *Alba* genes in rice are expressed at significantly higher levels under multivariate stresses and phytohormone treatments, but their exact role remains unclear.

### 3.9. Influence of Phytohormones on the Expression of the *OsAlba* Genes 

Phytohormones play an important role in mediating host responses to various biotic and abiotic stresses. ABA controls numerous physiological processes in plants and is best known for its regulatory role in abiotic stress tolerance. Under ABA treatment, *OsAlba1*, *OsAlba4*, and *OsAlba8* were found to be upregulated, whereas *OsAlba2*, *OsAlba3*, *OsAlba5*, *OsAlba6*, *OsAlba7*, and *OsAlba9* showed reduced expression. ABA has been reported to promote tolerance to desiccation under conditions of water deficit and hypersalinity [69]. The treatment with SA and JA showed upregulated expression of *OsAlba2*, *OsAlba3*, *OsAlba4*, *OsAlba5*, *OsAlba6*, *OsAlba7*, and *OsAlba9* but downregulated expression of *OsAlba1* and *OsAlba8*. The transcript abundance of *OsAlba7* was markedly induced under JA, whereas a mix pattern of expression was observed following treatment with SA and ABA, indicating its role in different physiological responses. Traditionally, SA and JA are known to be associated with resistance when plants are inflicted with biotrophic and necrotrophic pathogens [70,71]. However, the stress hormone ABA, better known for its role in the response to drought stress and in the maintenance of seed dormancy [72] has also been demonstrated to influence plant–pathogen interactions [73,74,75]. Altogether, the differential responses of *OsAlba* genes under various phytohormone treatments suggest the specific physiological roles of individual members of this family in rice.

### 3.10. Three-Dimensional Structure Prediction and Homology Modeling

The structural features of a protein predict its putative interactions and binding to various other molecules or ligands, and eventually provide its sequence–structure–function relationships. The sequence identity/similarity and accurate alignment between the template and a target protein leads to the prediction of 3D models. We selected the best template based on the QMEAN score value. The scores and the parameters of the selected templates for all OsAlba proteins are mentioned in Table 2.

**Figure 6:**
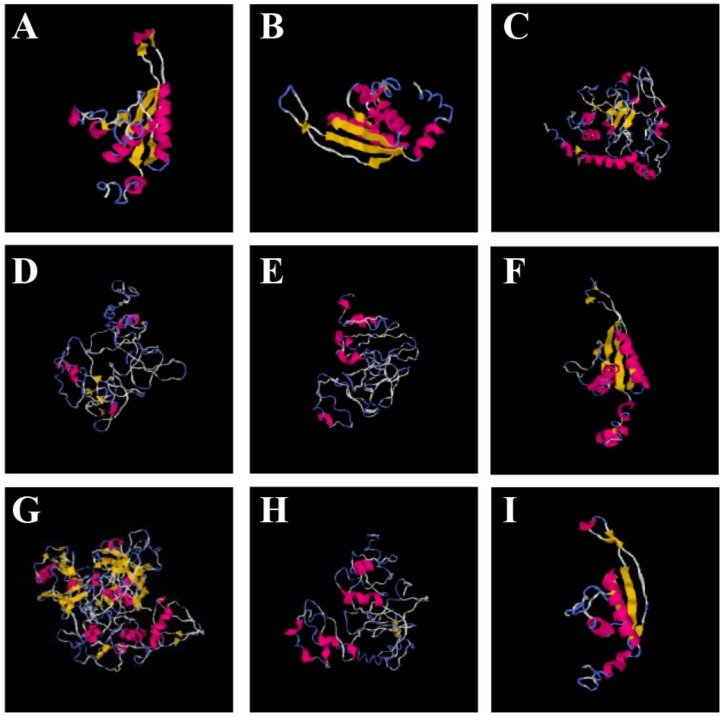
The 3-dimensional structures of OsAlba proteins. The best predicted models were selected from five models generated by I-TASSER. The secondary structure elements: α-helices (red), β-sheets (yellow), and coils (blue) are indicated for the predicted 3D structures of (**A**) OsAlba1; (**B**) OsAlba2; (**C**) OsAlba3; (**D**) OsAlba4; (**E**) OsAlba5; (**F**) OsAlba6; (**G**) OsAlba7; (**H**) OsAlba8, and (**I**) OsAlba9.

The amino acid sequences were submitted to LOMETS [76] to generate 3D structures. The 3D structures predicted for OsAlba proteins (Figure 6) were aligned to their respective templates in the TM-align server [77]. The predicted model was further analyzed in Chimera 1.2 [78], which showed different number of α-helices, β-strands, and coils (Figure 6). 

The OsAlba proteins showed the presence of 2–4 α-helices and 4–6 β-strands, while the coils were found in the range of 7–9, representing a high structural conservation. However, OsAlba7 showed very different structural components having 7 α-helices, 29 β-strands, and 37 coils (Table S5). The alignment with the template demonstrated a good structural match despite low sequence identities in some of the OsAlba proteins. The percentage sequence identity of the template with the query sequence ranged from 0.11 to 0.64, while the percentage sequence identity between the templates in the threading-aligned region and the query sequence remained between 0.09 and 0.80. The threading alignment coverage ranged between 0.33 and 0.98. The threading alignments had normalized Z-scores of >1.0, which suggests a significant alignment with the respective templates. The C-score for OsAlba4 was −4.03, while the score for OsAlba6 was −0.72. The values of other parameters (number of decoys and cluster density) remained in a reliable range (Table 3). 

The TM-score was found to be maximum for OsAlba6 (0.62) and minimum for OsAlba8 (0.28). The estimated RMSD was maximum for OsAlba7 and minimum for OsAlba9. The TM-score < 0.17 suggests a random similarity, and the TM-score > 0.5 indicates a model with correct topology. The TM-scores for the predicted template for all the OsAlba proteins were observed within an acceptable range, demonstrating the reliability of the models. The best aligned template and their Protein Database (PDB) IDs along with their sequence identities and query coverage of amino acid residues are mentioned in Table S6.

### 3.11. Structure–Function Relationship of OsAlba Proteins

The analysis of conserved patterns and of their structural components, as observed in the MSA profile of homologous sequences, provided potential information about the possible ligand-binding sites of the OsAlba proteins. The best predicted structural template was found to be IVM0B for OsAlba1, OsAlba6, and OsAlba9. The predicted template for OsAlba4, OsAlba5, and OsAlba8 was 4NL6A (Table S6). We identified the binding residues on the basis of the alignment between the template and the obtained OsAlba models. While OsAlba1, OsAlba2, OsAlba3, OsAlba6, and OsAlba9 showed binding affinity for arginine and nucleic acids and OsAlba1, OsAlba3, and OsAlba6 were predicted to bind both RNA and DNA, OsAlba2 had binding affinity for RNA only. The RNA-binding properties of the Alba proteins in vivo, apart from their DNA-binding ability in a histone-like manner, has been studied in archaea [4,6,12,13]. Earlier, the binding of the Alba proteins to nucleic acids was reported from other species [14]. OsAlba4, OsAlba5, OsAlba7, and OsAlba8 showed no binding to nucleic acids, but showed an affinity for ligands such as KA, PHR, Mg, and chlorophyll-a (Figure S2). Previous studies had reported the function of Alba in transcriptional regulation through nucleic acid-binding [79]. The binding of the OsAlba proteins with different ligands suggests their various functions in different environmental conditions. The stress-responsive function of OsAlba1 has previously been established in various abiotic stress treatments [53]. GO annotation further suggests various roles of the Alba family proteins (Table S7). While OsAlba1, OsAlba2, and OsAlba9 were found to have a putative role in DNA-packaging and chromosome organization, OsAlba3 was predicted to function in transcriptional regulation and gene expression. OsAlba5 was predicted to help in DNA replication, whereas OsAlba7 was predicted to function in proteolysis. OsAlba4 and OsAlba8 exhibited putative roles in oxido–reductive and metabolic processes, respectively, indicating their similarity in structure as well as function. 

## 4. Conclusions

In the present study, we identified 68 *Alba* genes from 9 different species across the plant kingdom and evaluated the gene structure, phylogenetic relationships, upstream regulatory elements, conserved motifs, and their subsequent transcriptional regulation through miRNA target sequences. A number of CAREs were found in the regulatory sequences upstream of the *Alba* genes, suggesting their expression through a complex regulatory scheme. The transcript profile of the *OsAlba* genes showed their distinct tissue-specific expression, indicating their specific roles in rice. The *OsAlba* transcript profiles under dehydration, hypersalinity, heat, cold, and phytohormone treatments indicate that most *OsAlba* genes might play a crucial role for stress adaptation. Additionally, the distinct subcellular localization of the OsAlba proteins and their homologs in other plant species suggests their organelle-specific biological function. The structural features of the Alba proteins and their evolutionary relationships aided in predicting their putative functions. The results altogether will not only facilitate the understanding of the molecular mechanisms of stress-responsive adaptation in rice but will also give new insights on the role of the Alba proteins in plants, in general.

## Figures and Tables

**Table 1:** Identification and chromosomal location of plant-specific *Alba* genes.

Plants	Name	Chromosome No.	Gene Location	Exons	Length (bp)	Number of aa	MW (kDa)	Iso Electric Point	Identity/*e*-Value	Accession Numbers
*Chlamydomonas*	*CreAlba1*	9	864278..866101 forward	5	1824	143	15.12	5.02	100/5 × 10^−97^	XP_001695217.1
*Physcomitrella* moss	*PpAlba1*	12	13891844..13896427 reverse	1	4584	131	14.7	5.14	100/1 × 10^−90^	XP_001768991.1
	*PpAlba2*	20	9435522..9440461 forward	8	4940	265	28.79	9.76	100/3 × 10^−171^	XP_001767525.1
	*PpAlba3*	23	6029976..6034958 reverse	8	4983	268	29.2	10.06	100/0	XP_001753647.1
	*PpAlba4*	24	3314088..3317325 forward	8	3238	266	29.07	10.2	100/0	XP_001755565.1
*Amborella*	*AmtrAlba1*	scaffold00002	8145087..8147916 forward	5	2830	131	14.2	9.46	100/4 × 10^−91^	XP_020520141.1
	*AmtrAlba2*	scaffold00017	5252778..5262210 forward	8	9433	263	28.48	10.06	100/0	XP_006847137.1
	*AmtrAlba3*	scaffold00067	2229721..2240829 reverse	9	11,109	303	33.94	4.89	100/0	ERN18878.1
	*AmtrAlba4*	scaffold00104	1833918..1840085 reverse	5	6168	132	14.69	5.67	100/4 × 10^−90^	XP_006837817.1
Grape	*VvAlba1*	5	6754914..6756137 forward	5	1224	131	14.24	9.6	100/6 × 10^−89^	XP_002280347.1
	*VvAlba2*	7	9926246..9934777 forward	8	8532	244	27.07	10.48	100/5 × 10^−171^	XP_010652481.1
	*VvAlba3*	7	18184062..18194607 forward	10	10,546	282	31.19	10.24	100/0	CBI21553.3
	*VvAlba4*	9	6407205..6412879 forward	5	5675	129	14.39	5.47	100/3 × 10^−88^	CBI36290.3
	*VvAlba5*	11	4382031..4385728 forward	5	3698	130	14.55	5.28	100/2 × 10^−89^	XP_002285115.1
	*VvAlba6*	18	1752391..1758060 forward	8	14,972	257	28.3	10.4	100/0	CBI18930.3
	*VvAlba7*	18	19508351..19517807 forward	8	9457	259	28.34	10.13	100/0	XP_002264932.1
Chickpea	*CaAlba1*	1	9204378..9202500 reverse	5	1879	134	14.74	5.23	100/2 × 10^−90^	XP_004486682.1
	*CaAlba2*	4	4931675..4930115 reverse	5	1561	135	14.71	9.46	100/1 × 10^−91^	XP_004495605.1
	*CaAlba3*	5	46947757..46950898 forward	8	3142	246	27.23	10.14	100/1 × 10^−171^	XP_004502885.1
	*CaAlba4*	5	46952324..46956941 forward	9	4618	312	34.58	10.24	100/0	XP_004502886.1
	*CaAlba5*	5	46958526..46962753 reverse	8	4228	241	26.81	9.82	100/4 × 10^−169^	XP_004502888.1
	*CaAlba6*	6	50716068..50719107 reverse	5	3040	133	14.46	5.42	100/2 × 10^−89^	XP_004506910.1
	*CaAlba7*	8	786966..785427 reverse	5	1540	123	13.48	9.35	100/3 × 10^−81^	XP_004511396.1
*Arabidopsis*	*AtAlba1*	1	7004879..7007651 reverse	10	2773	315	33.76	9.92	100/0	NP_564108.1
	*AtAlba2*	1	10223266..10224727 reverse	5	1462	130	14.57	5.31	100/5 × 10^−87^	NP_564325.1
	*AtAlba3*	1	28528077..28530790 reverse	8	2714	350	37.38	9.78	100/0	NP_565124.1
	*AtAlba4*	2	14426203..14427383 forward	5	1181	130	14.62	5.28	100/3 × 10^−87^	NP_565781.1
	*AtAlba5*	3	1255526..1256869 reverse	5	1344	164	17.66	5.79	100/4 × 10^−111^	NP_187113.1
	*AtAlba6*	3	2223001..2225254 reverse	8	2254	405	42.5	9.15	100/0	NP_187359.2
Rice	*OsAlba1*	1	3742694..3744539 reverse	5	1684	152	16.26	5.1	100/4 × 10^−101^	NP_001042157.1
	*OsAlba2*	2	5740408..5742520 reverse	5	2113	145	15.51	5.17	100/6 × 10^−97^	BAD15498.1
	*OsAlba3*	3	30119573..30123252 reverse	9	3645	320	35.39	9.81	100/0	EEE59881.1
	*OsAlba4*	3	3565365..3561501 reverse	8	3770	309	33.21	9.99	100/0	NP_001049072.1
	*OsAlba5*	4	21232538..21235466 reverse	7	2922	262	28.51	9.06	100/0	CAD41015.3
	*OsAlba6*	6	23821372..23823339 forward	5	1870	132	14.61	5.5	100/6 × 10^−89^	NP_001058008.1
	*OsAlba7*	9	21343532..21346652 reverse	17	6507	671	71.68	8.59	100/0	EEE70143.1
	*OsAlba8*	11	3297930..3303336 forward	8	5111	302	34.46	9.06	100/0	AAX92995.1
	*OsAlba9*	12	18351588..18353679 reverse	4	2092	124	14.03	8.96	100/1 × 10^−83^	EEC69301.1
Maize	*ZmAlba1*	1	17932570..17953959 reverse	11	21,390	443	49.15	8.59	100/0	DAA43763.1
	*ZmAlba2*	1	197014554..197016807 reverse	5	2254	95	10.52	9.2	100/2 × 10^−62^	DAA48353.1
	*ZmAlba3*	1	203886863..203889397 reverse	9	2535	435	47.21	9.53	100/0	DAA48594.1
	*ZmAlba4*	1	273633103..273636648 reverse	8	3546	318	34.4	9.85	100/0	NP_001140948.1
	*ZmAlba5*	2	52865684..52870403 forward	8	4720	259	28.5	9.68	100/2 × 10^−180^	NP_001140391.1
	*ZmAlba6*	2	138609879..138620022 reverse	8	10,144	276	30.98	9.31	100/0	NP_001146602.1
	*ZmAlba7*	3	12031159..12039337 forward	5	8179	146	15.79	5.06	100/1 × 10^−98^	NP_001141319.1
	*ZmAlba8*	3	140869717..140869074 reverse	3	3345	160	18.03	6.94	100/1 × 10^−112^	XP_008676084.1
	*ZmAlba9*	4	25351731..25363303 reverse	8	11,573	262	29.58	9.31	100/0	AFW60929.1
	*ZmAlba10*	4	232536528..232538464 forward	5	1937	139	15.03	5.75	100/2 × 10^−93^	NP_001143331.1
	*ZmAlba11*	5	8422556..8425967 forward	8	3412	355	38.09	9.77	100/0	XP_008644524.1
	*ZmAlba12*	7	100322879..100327949 reverse	7	5071	348	47.42	10.07	100/1 × 10^−33^	DAA51661.1
	*ZmAlba13*	8	17424473..17428831 reverse	5	4359	146	15.87	5.19	100/7 × 10^−99^	ACN31274.1
	*ZmAlba14*	8	73390426..73394062 reverse	10	3637	265	30.02	8.47	100/0	AFW81235.1
	*ZmAlba15*	8	79903859..79905084 forward	5	1226	174	18.49	5.7	100/1 × 10^−120^	XP_008656129.1
	*ZmAlba16*	9	85118424..85120875 reverse	5	2452	122	13.91	9.22	100/2 × 10^−81^	AFW87068.1
	*ZmAlba17*	9	153687377..153692790 reverse	8	5414	305	33.26	9.69	100/0	NP_001132775.1
	*ZmAlba18*	10	13160976..13162521 forward	5	1546	198	21.69	4.74	100/5 × 10^−133^	XP_008662548.1
	*ZmAlba19*	10	65669734..65672121 reverse	7	2388	269	28.91	9.86	100/0	AFW57019.1
	*ZmAlba20*	10	125226158..125230941 forward	7	4784	173	19.16	8.57	100/2 × 10^−124^	AFW58594.1
*Sorghum*	*SbAlba1*	1	7634252..7637528 forward	8	3277	345	36.9	9.87	100/0	XP_002463920.1
	*SbAlba2*	1	76251059..76254951 forward	8	3893	299	32.42	9.89	100/0	KXG40112.1
	*SbAlba3*	2	66308102..66311291 reverse	8	3190	267	29.82	9.82	100/0	XP_002462756.1
	*SbAlba4*	3	4567538..4570747 forward	5	3210	147	15.76	5.17	100/5 × 10^−99^	XP_002455102.1
	*SbAlba5*	4	7088829..7090849 reverse	5	2021	141	15.24	5.5	100/4 × 10^−95^	XP_002453505.1
	*SbAlba6*	5	4826765..4832055 forward	8	5291	275	30.93	9.39	100/0	XP_002449052.1
	*SbAlba7*	6	44833619..44836936 reverse	8	3318	261	28.38	9.57	100/0	XP_002447839.1
	*SbAlba8*	8	46970489..46972066 reverse	5	1578	190	20.73	4.87	100/4 × 10^−128^	XP_002443196.1
	*SbAlba9*	9	6694750..6696439 reverse	5	1690	134	14.3	5.43	100/7 × 10^−90^	XP_002440693.1
	*SbAlba10*	10	52387417..52389679 forward	5	2263	129	14.39	5.29	100/1 × 10^−86^	XP_002437246.1

**Table 2:** Secondary structure validation for OsAlba proteins by I-TASSER.

S. No.	GI Number	Type	TM-Score	RMSD
1	115434798	OsAlba1	0.49 ± 0.15	8.8 ± 4.6
2	46390081	OsAlba2	0.50 ± 0.15	8.4 ± 4.5
3	62701922	OsAlba3	0.34 ± 0.11	14.7 ± 3.6
4	115450943	OsAlba4	0.28 ± 0.09	16.3 ± 3.0
5	XP_006652291.1	OsAlba5	0.29 ± 0.09	15.8 ± 3.2
6	XP_015643177.1	OsAlba6	0.62 ± 0.14	6.0 ± 3.7
7	222642011	OsAlba7	0.34 ± 0.11	16.6 ± 2.9
8	62701922	OsAlba8	0.30 ± 0.10	15.6 ± 3.3
9	218186874	OsAlba9	0.57 ± 0.15	6.8 ± 4.1

RMSD: root-mean-square deviation between residues.

**Table 3:** Parameters for 3D structure prediction of OsAlba proteins.

S. No.	Type	GI Number	Score	No. of Decoys	Cluster Density
1	OsAlba1	115434798	−1.83	3476	0.0607
2	OsAlba2	46390081	−1.73	3848	0.0657
3	OsAlba3	62701922	−3.41	621	0.0170
4	OsAlba4	115450943	−4.03	626	0.0091
5	OsAlba5	XP_006652291.1	−4.00	621	0.0093
6	OsAlba6	XP_015643177.1	−0.72	7113	0.1765
7	OsAlba7	222642011	−3.36	171	0.0100
8	OsAlba8	62701922	−3.81	621	0.0113
9	OsAlba9	218186874	−1.18	6399	0.1204

## Supplementary Materials

The following are available online at http://www.mdpi.com/2073-4425/9/4/183/s1, Table S1: The primer sequences of *OsAlba* gene for qRT-PCR, Figure S1: Conserved motif arrangement in the plant-specific Alba proteins. The font size of amino acid sequences of each motif represents the frequency of the respective amino acid, Table S2: Subcellular localization of plant-specific Alba proteins, Table S3: *cis*-acting regulatory elements in plant-specific Alba promoters, Table S4: Identification of miRNA targets in plant-specific *Alba* genes, Table S5: Secondary structure elements in OsAlba proteins, Table S6: Templates for 3D structure prediction of OsAlba proteins, Figure S2: Prediction of ligands binding to OsAlba proteins. OsAlba proteins binding to (A) RNA, (B) Arginine (ARG), (C) Chlorophyll-a, (D) KA and PHR (E) DNA, and (F) Magnesium (MG), Table S7: GO annotation for OsAlba proteins.
