# Supplementary material for: Genome-Wide Identification of the Alba Gene Family in Plants and Stress-Responsive Expression of the Rice Alba Genes
Source: Genes (Basel). 2018 Mar 28;9(4):183. doi: 10.3390/genes9040183 (PMC5924525; doi:10.3390/genes9040183)
Supplement: Supplementary file 1 [file genes-09-00183-s001.zip › Supplementary files/Figure S1.pdf]

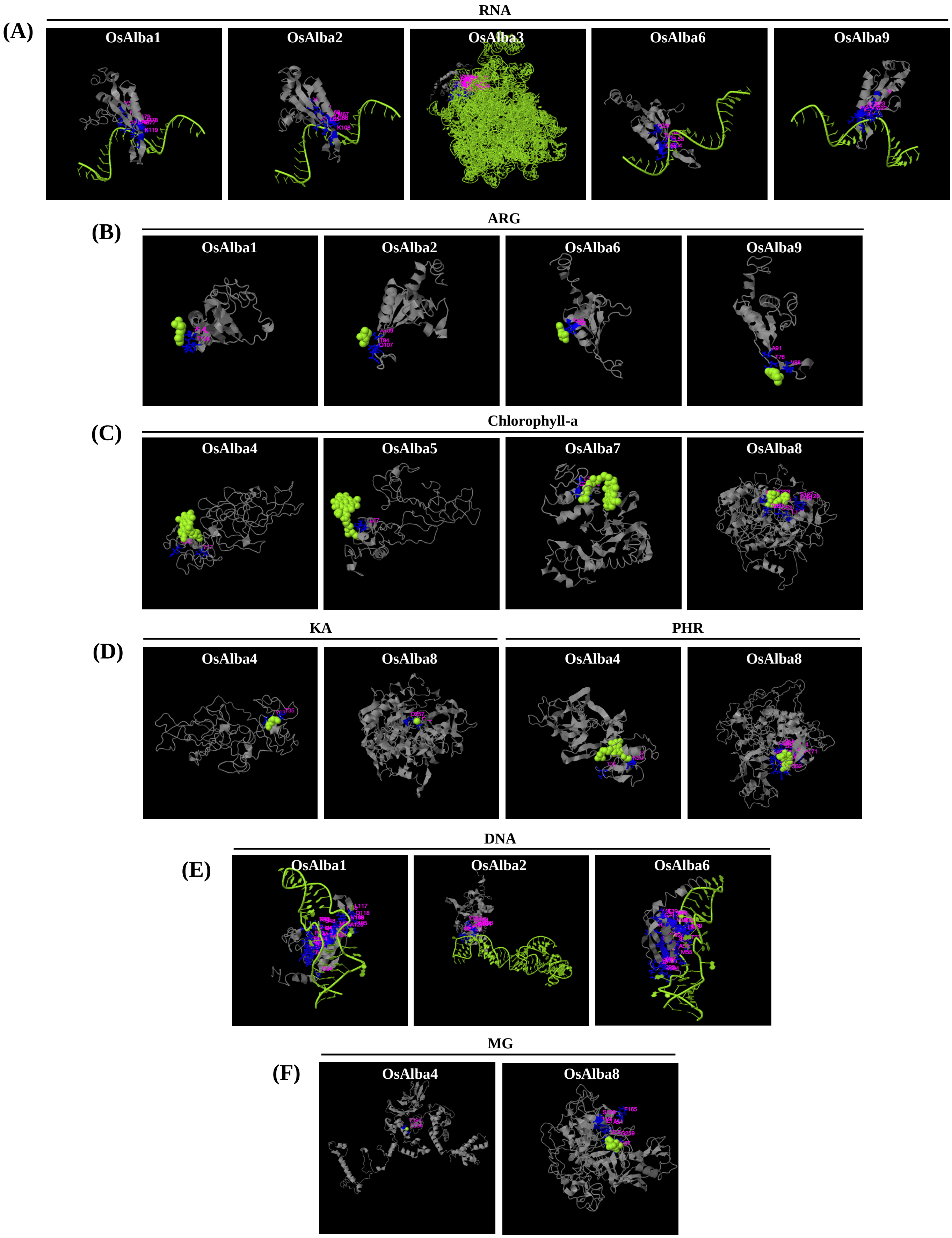

Figure S1. Prediction of ligands binding to OsAlba proteins. OsAlba proteins binding to **(A)** RNA, **(B)** Arginine (ARG), **(C)** Chlorophyll-a, **(D)** KA and PHR **(E)** DNA and **(F)** Magnisium (MG).
