# Supplementary material for: Genome-Wide Identification of the Alba Gene Family in Plants and Stress-Responsive Expression of the Rice Alba Genes
Source: Genes (Basel). 2018 Mar 28;9(4):183. doi: 10.3390/genes9040183 (PMC5924525; doi:10.3390/genes9040183)
Supplement: Supplementary file 1 [file genes-09-00183-s001.zip › Supplementary files/Table S5.pdf]

Table S5. Secondary structure elements in OsAlba proteins.

S No.	Protein name	α- Helix	β- Sheets	Coils
1	OsAlba1	4	5	8
2	OsAlba2	4	5	7
3	OsAlba3	2	5	7
4	OsAlba4	2	6	7
5	OsAlba5	3	6	8
6	OsAlba6	3	4	7
7	OsAlba7	7	29	37
8	OsAlba8	6	6	11
9	OsAlba9	5	5	9
