# Supplementary material for: Genome-Wide Identification of the Alba Gene Family in Plants and Stress-Responsive Expression of the Rice Alba Genes
Source: Genes (Basel). 2018 Mar 28;9(4):183. doi: 10.3390/genes9040183 (PMC5924525; doi:10.3390/genes9040183)
Supplement: Supplementary file 1 [file genes-09-00183-s001.zip › Supplementary files/Table S6.pdf]

Table S6. Templates for 3D structure prediction of OsAlba proteins.

**S. No.**	**Protein**	**PDB Hit**	**IDEN**	**Cov**	**Z-score**
1	OsAlba1	1VM0B	0.848	0.651	1.74
2	OsAlba2	2Q3VB	0.828	0.676	1.77
3	OsAlba3	3J65R	0.090	0.903	1.02
4	OsAlba4	4NL6A	0.037	0.851	1.24
5	OsAlba5	4NL6A	0.111	0.836	1.25
6	OsAlba6	1VM0B	0.838	0.735	2.06
7	OsAlba7	4PPHA	0.181	0.544	1.65
8	OsAlba8	4NL6A	0.057	0.851	1.51
9	OsAlba9	1VM0B	0.510	0.790	1.78

IDEN- percentage sequence identity in the structurally aligned region.

Cov- coverage of alignment by TM align.

Z-score **-** alignment with a normalized z-score greater than 1 means good alignment.
