# Supplementary material for: Genome-Wide Identification of the Alba Gene Family in Plants and Stress-Responsive Expression of the Rice Alba Genes
Source: Genes (Basel). 2018 Mar 28;9(4):183. doi: 10.3390/genes9040183 (PMC5924525; doi:10.3390/genes9040183)
Supplement: Supplementary file 1 [file genes-09-00183-s001.zip › Supplementary files/Table S7.pdf]

Table S7. GO annotation for OsAlba proteins.

	Molecular Process	Biological Process	Cellular component
**OsAlba1**	RNA, DNA Binding	DNA packaging, chromosome organisation	Nuclear, Chromosome
**OsAlba2**	RNA, DNA Binding	DNA packaging, chromosome organisation	Nuclear, Chromosome
**OsAlba3**	RNA, DNA Binding	Regulation of Transcription, gene expression	Intracellular organelle
**OsAlba4**	ATP binding	Oxido-reuctive process	Nuclear
**OsAlba5**	RNA Binding	DNA replication	RNA reductase
**OsAlba6**	ATP binding	Not predicted	Chromosome, Cytosol
**OsAlba7**	Endopeptidase activity	Proteolysis	Golgi, extracellular region
**OsAlba8**	Acetyl transferase, choline binding	Choline metabolism	Nuclear envelop
**OsAlba9**	RNA, DNA Binding	Chromosome condensation	Chromosome
