# Supplementary material for: Genome-Wide Identification of the Alba Gene Family in Plants and Stress-Responsive Expression of the Rice Alba Genes
Source: Genes (Basel). 2018 Mar 28;9(4):183. doi: 10.3390/genes9040183 (PMC5924525; doi:10.3390/genes9040183)
Supplement: Supplementary file 1 [file Figure S2.pdf › Supplementary files/Figure S2.pdf]

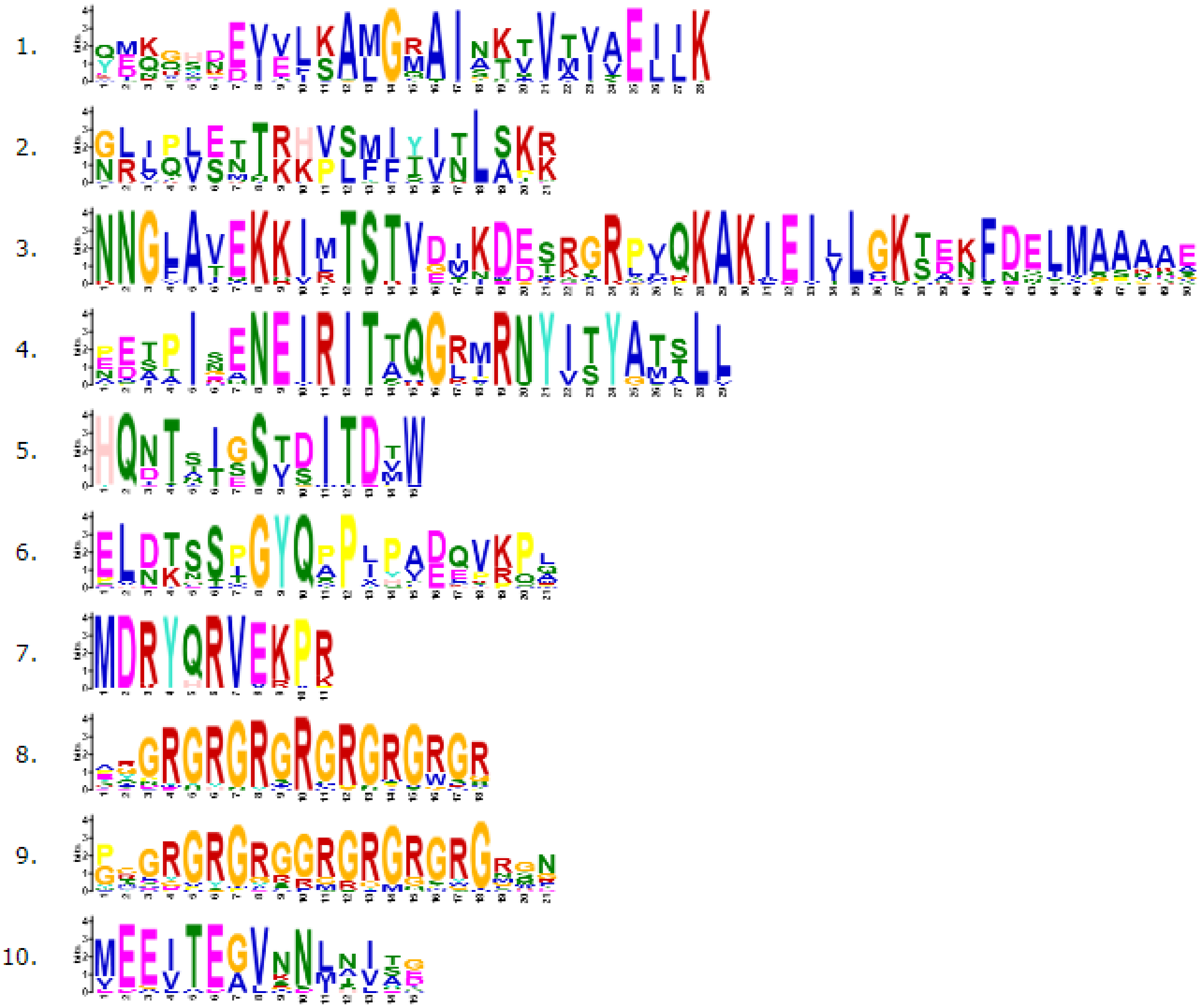

Figure S2. Conserved motifs arrangement in the plant specific Alba proteins. Amino acid sequences of each motif. The font size represents the frequency of the respective amino acid.
